# Development of Cell-SELEX Technology and Its Application in Cancer Diagnosis and Therapy

**DOI:** 10.3390/ijms17122079

**Published:** 2016-12-10

**Authors:** Man Chen, Yuanyuan Yu, Feng Jiang, Junwei Zhou, Yongshu Li, Chao Liang, Lei Dang, Aiping Lu, Ge Zhang

**Affiliations:** 1 Institute of Integrated Bioinformedicine and Translational Science, School of Chinese Medicine, Hong Kong Baptist University (HKBU), Hong Kong 999077, China; chen131195@163.com (M.C.); yu.yy01@hotmail.com (Y.Y.); jiangfenghz@163.com (F.J.); Waynebo@163.com (J.Z.); yongshuli000@163.com (Y.L.); liangchao512@163.com (C.L.); danglei_hkbu@163.com (L.D.); 2 Institute of Precision Medicine and Innovative Drug Discovery, HKBU (Haimen) Institute of Science and Technology, Haimen 226100, China; 3 Shenzhen Lab of Comninatorial Compounds and Targeted Drug Delivery, HKBU Institute of Research and Continuing Education, Shenzhen 518000, China; 4 The State Key Laboratory Base of Novel Functional Materials and Preparation Science, Faculty of Materials Science and Chemical Engineering, Ningbo University, Ningbo 315211, China

**Keywords:** SELEX, aptamer, cell-SELEX, diagnosis, therapy, cancer

## Abstract

SELEX (systematic evolution of ligands by exponential enrichment) is a process involving the progressive isolation of high selective ssDNA/RNA from a combinatorial single-stranded oligonucleotide library through repeated rounds of binding, partitioning and amplification. SELEX-derived single-stranded DNA/RNA molecules, called aptamers, are selected against a wide range of targets, including purified proteins, live cells, tissues, microorganisms, small molecules and so on. With the development of SELEX technology over the last two decades, various modified SELEX processes have been arisen. A majority of aptamers are selected against purified proteins through traditional SELEX. Unfortunately, more and more evidence showed aptamers selected against purified membrane proteins failed to recognize their targets in live cells. Cell-SELEX could develop aptamers against a particular target cell line to discriminate this cell line from others. Therefore, cell-SELEX has been widely used to select aptamers for the application of both diagnosis and therapy of various diseases, especially for cancer. In this review, the advantages and limitations of cell-SELEX and SELEX against purified protein will be compared. Various modified cell-SELEX techniques will be summarized, and application of cell-SELEX in cancer diagnosis and therapy will be discussed.

## 1. Introduction

Aptamers are single-stranded DNAs or RNAs that fold into unique 3D structures for interacting with specific targets. Compared with other ligands, aptamers’ small size, facile chemical synthesis, excellent chemical stability, versatility in structural design and engineering and low immunogenicity enable their widely use in cancer imaging and therapy applications [1]. In recent years, there are growing numbers of aptamers being exploited and applied in various areas including disease diagnosis, clinical therapy, analytical chemistry, food safety, bio-sensing and environmental toxicity detection [2].

SELEX (systematic evolution of ligands by exponential enrichment) technology, established by Tuerk and Ellington in 1990, is the method used to identify aptamers with high affinitive and selective [3,4]. With the optimization and development of SELEX methodology, a wide range of targets could be directly used in the selection, including small molecules, proteins, viruses, bacteria, live cells, and even tissues [5]. Proteins are the most common targets used in SELEX for the identification of aptamers [6]. However, it is rather difficult to obtain enough high purity recombinant human proteins with native conformation from various in vitro expression systems, especially for transmembrane proteins and intracellular proteins. Therefore, in order to solve these problems, researchers have been working on developing new methods for aptamer selection.

In 1998, Morris and Jensen [7] firstly used human red blood cell membranes as a complex mixture target to select aptamers through cell-based SELEX methodology (cell-SELEX), which provided an in vitro protocol for isolating high affinitive aptamers specifically against complex mixture of potential targets. Unlike other SELEX methods, cell-SELEX selects aptamer against a whole cell, so molecular targets on the cell surface are in their native state and would represent their natural folding structures [8]. In the past two decades, aptamers have been developed for a wide variety of live cells and other complex systems, especially for live cancer cells [9].

In this review, we will first bring a comprehensive description of the development of cell-SELEX methodology and explain its advantages and limitations by comparing with traditional SELEX and protein-SELEX. Then we will expound the cell-SELEX strategy and procedure in details and talk about recent progress in the exploitation of new technologies based on cell-SELEX. Finally, an overview of the application and prospects of aptamers selected by cell-SELEX in cancer diagnosis and therapy will be given, which would be helpful for understanding and facilitating the application of cell-SELEX technology in future.

## 2. Advantages and Limitations of Cell-SELEX

### 2.1. Advantages of Cell-SELEX

The emergence of cell-based screening methods greatly enriched screening targets and expands the potential applications of aptamers. Cell-SELEX has been the first choice in developing aptamers to recognize particular biomarkers on cancer cell surface for the application of both diagnostic and therapeutic purpose. For developing membrane protein aptamers against particular diseases using traditional protein-SELEX, prior knowledge of protein targets is necessary in the first place. Furthermore, enough recombinant membrane proteins with high purity would be needed. However, because of the post-translational modifications, membrane proteins expressed in prokaryotic or some eukaryotic systems often cannot fold into the correct 3D structure that is formed under physiologic conditions. This causes the low solubility and low yield of membrane proteins expressed in vitro expression systems, which limit their application [5]. Cell-SELEX overcomes the difficulties in obtaining purified recombinant membrane proteins [10]. In cell-SELEX, aptamers are developed against molecules on the cell surface without requirement for prior knowledge of the molecular targets [11]. Therefore, protein purification is also not necessary in prior to the selection.

Large transmembrane molecules are functionally important molecules involved in many biological processes, such as signal transduction, cell adhesion and migration, cell–cell interactions, and communication between the intra- and extra-cellular environments [12]. Yet the membrane proteins aptamers developed through protein-based SELEX may not be able to selectively recognize and interact with their corresponding targets in vitro, which would result in failure of the bio-medical application [5]. In cell-SELEX, all molecules on the cell surface are in their native states and would therefore represent their natural folding structures and distribution. All post-translational modifications are left intact for proteins, and so aptamers will bind to the real folded conformation [8]. Therefore, cell-SELEX eliminates the risk that identified aptamers would only bind to the purified proteins but could not recognize the native form of the proteins on living cells.

Moreover, cell-SELEX also can be used to discovery new biomarkers in particular cancer cells surface. Prabodhika et al. [13] presented a strategy for identifying proteins with expression level changed in a diseased cell using cell specific aptamers. They used the selected aptamers that showed different recognition patterns with different cell lines of leukemia to capture and enrich the target receptor proteins. And followed by mass spectrometry, they recognized the receptor was the membrane-bound immunoglobin heavy mu chain in Burkitt’s lymphoma cells [13]. This study, which discovered new biomarkers in particular cancer cells, demonstrates that specific aptamers could be developed by cell-SELEX and in turn used as probes to identify target. Cell-SELEX strategy, as well as the aptamers selected from cell-SELEX offer valuable tools to isolate the disease-specific protein targets and facilitate the discovery of clinically important biomarkers. They would open a door for the development of ‘‘personalized’’ medicine and novel biological probes technologies [14].

### 2.2. Limitations of Cell-SELEX

Although cell-SELEX has a great potential in the biomedical field, several technical limitations remain and must be addressed in the following optimizations. Firstly, cell condition is rather important in aptamer selection. Presence of dead cells in a suspension will lead to non-specific uptake and binding of oligonucleotides which would be a negative impact on whole selection process. Such methods may be possible solutions to remove dead cells and help to decrease possibility of nonspecific aptamers obtained through cell-SELEX. For example, for this purpose, Raddatz et al. [15] implemented fluorescence-activated cell-sorting (FACS) in the cell-SELEX procedure to separate aptamers bound to vital suspension cells (Ramos Burkitt’s lymphoma B cells). Aptamers were incubated together with vital and dead cells and only aptamers bound to calcein-AM-stained vital cells were collected. Meltem et al. [16] also established a method to remove dead cells from the cell suspension. The cell suspension was centrifuged after the detachment of cells with EDTA to discard a large number of dead cells, which remained in the supernatant. Thereafter, the remaining dead cells were isolated by magnetic depletion using dead cell removal microbeads. The amount of dead cells could be reduced to 5.2% using the optimized method. All these approaches efficiently optimized the selection strategies for generation of cell-specific aptamers.

Since the cell surface components are very complex, additional counter selections against other non-target cells are essential in order to improve the specificity of aptamers, and therefore the operation is more complex [5]. This might have a negative economic impact on selection as increasing the time and cost will be required. Automated SELEX could generate aptamers with the required qualities within several days and multiple targets could be handled at the same time which is rather efficient and labor free [17]. Moreover, selection cycles can be automatically conducted without any requirements of direct intervention steps during the whole selection process [18]. As yet, various attempts on automatic in vitro selections have been made in the past few years. Companies of Aptasol (York, UK) and Vivonics (Sudbury, MA, USA) have been committed to the development of rapid selection systems, such as automated high-throughput selection and one-step selection [19]. With flow cytometry and high throughput sequencing technology successfully adopted into the cell-SELEX procedure, it is believed that cell-SELEX technology will be developed to shorten the selection period, improve success rate, and accelerate high-affinity aptamer identification in the near future.

Another difficult step is how to successfully identify of the target of the aptamers generated by cell-SELEX. On the one hand, cells are complex targets to aptamers and the cell surface components are very complex. On the other hand, some aptamers are not only capable of selectively binding to the membrane protein in the target cell surface, and even internalizing into the target cells [20]. Thses increase the difficulty for target identification. Biomarker discovery is also a pressing task in molecular medicine. Tan’s laboratory developed their own procedure for target membrane protein identification, and they have identified several novel protein targets using this approach [9]. We hope to have more methods applied to discover the target of the aptamers selected from cell-SELEX in the future.

Furthermore, it is known that the cell surface carries a net negative charge and thus repulsion would occur between the DNA polyanion and the cell surface [21]. Therefore, it would be difficult to generate nucleic acid aptamers binding to cell surface. On the other hand, in order to avoid membrane protein in the cell surface being covered, target cells for cell-SELEX can not be fixed, so the separation efficiency of binding complex with unbound nucleic acids is low [22]. Currently, there is still no effective method to overcome these problems, but more efforts would be made to optimize the cell-SELEX technology to eliminate the negative effect of these limitation. 

## 3. Cell-SELEX Strategy and Procedure

**Figure 1:**
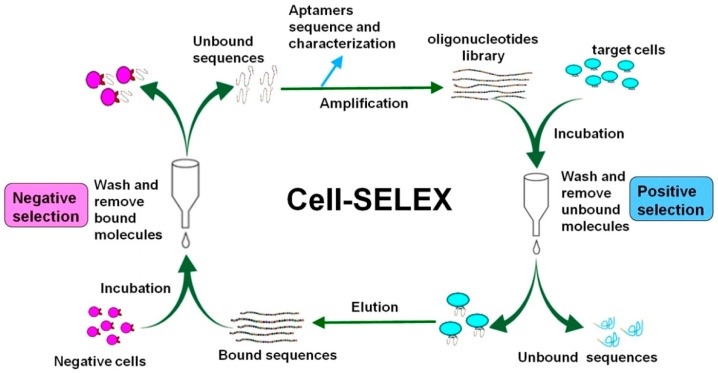
Schematic representation of aptamer selection using the cell-SELEX (systematic evolution of ligands by exponential enrichment) strategy.

The main steps of cell-SELEX are similar to traditional SELEX, which includes incubation, partitioning, and amplification. More details are illustrated in Figure 1. To generate aptamers that can specifically target cancer cells, the protocol of cell-SELEX includes positive selection and negative selection. The step of negative selection is necessary to remove sequences which binding to normal cells and improve the specificity of candidate aptamers.

Aptamers are chemically synthesized, short single-stranded DNA (ssDNA) or RNA molecules. Here we take ssDNA aptamers as an example to describe the process of cell-SELEX. Firstly, a single stranded oligonucleotide library which has a high diversity of random sequences are synthesized and incubated with the target cells. After washing, the DNA sequences bound to the target cell surface are eluted by heating cell-ssDNA complexes at 95 °C from the cells and collected by centrifugation. And the recovered pool is incubated with the negative control cells (control cells that do not express the target biomarker). All ssDNA sequences that show binding to the negative control cells are removed. Unbinding sequences are amplified by PCR using biotinylated reverse primer, leading to the enrichment of specific binders to the target. Streptavidin magnetic beads are used to capture the biotinylated antisense strand and unlabeled sense ssDNA is separated by NaOH. In general, a corresponding steady increase in binding affinity of the aptamer candidates is observed through the selection rounds increasing. The enrichment of the selected pools is monitored by flow cytometry binding assays. Finally, the enriched pools are sequenced and representative DNA aptamers are chosen for subsequent characterization [8]. Several factors need to be taken into consideration in cell-SELEX.

(1) Design the oligonucleotides library for SELEX. Four factors are involved: type of randomization, the length of the random sequence region, the chemistry of the pool, and the utility of the constant regions [6]. Aptamers can be RNA or single-strand DNA. The original report of SELEX used a randomized RNA pool, RNA SELEX generally involves in vitro transcription. It is more complex than the amplification process of DNA SELEX. So that following studies gradually used single-stranded DNA pools to generate DNA aptamers for a wide variety of targets instead. In general, RNA has better diversity in the fold due to the 2’OH group, whereas DNA is more stable, cheaper and easy production [23]. No significant differences in specificity or binding abilities have been observed between these two types. Nucleic acid libraries offer a great diversity of species and the ease of screening. Length of the random region of the starting library is normally between 20 to 40 bp [11]. Modified nucleotides could be also included in the library, which may greatly broaden the range of possible sequences and probably enhance their in vivo stability or nuclease resistance [24]. The design of the conserved primer regions is also important as improper design would introduce unspecific products in PCR. It can be designed using appropriate software such as Integrated DNA Technologies according to standard primer design considerations, including reasonable annealing temperature, proper G-C content, no primer heterodimers and primer self-dimers [8].

(2) Cell-SELEX uses whole live cells as targets and therefore good cell culture maintenance is very important. Cell overgrowth leads to more cell death, and probably causes an alteration in cell morphology and protein expression. The elimination of dead cells can significantly enhance the success rate of screening. The choice of cell lines depends on the purpose of the selection and what goals to achieve. Cell-SELEX has commonly been performed using cultured cancer cell lines, such as human hepatocarcinoma cell [25], prostate cancer cell [26], and cancer stem cells [27], etc. With these cell lines, aptamers that can differentiate between two different cancers or between cancer and normal cells have been generated [8].

(3) Negative SELEX is used to improve the selectivity of aptamers by excluding a portion oligonucleotide which can bind to similar target cells. The negative followed by the positive selection steps are carried out to filter out sequences against the molecules existing on the surface of both the target and control cell lines. These steps should be repeated several times to enrich the aptamer pool for target cells [28].

(4) Proper separation methods need to be chosen in ssDNA regeneration. There are several methods reported to generate ssDNA from double-stranded PCR products, including asymmetric PCR, denaturing high-performance liquid chromatography (DHPLC) method, lambda exonuclease digestion, size separation by denaturing urea–polyacrylamide gel derived from unequal primers with chemical modification and magnetic separation with streptavidin-coated beads. Asymmetric PCR use different amount of forward and reverse primers [29]. When the primer in limiting amount has been used up, an excess of ssDNA will be produced in each cycle. However, as an unequal molar ratio of the two primers is used in the PCR reaction, the diversity of ssDNA in the enriched oligonucleotide pools may be reduced. In the denaturing high-performance liquid chromatography (DHPLC) method [30], one of the two primers used in a PCR is biotinylated and another is normal. This method used the DNA wave fragment analysis system that incorporated denaturing reverse-phase ion pair high-performance liquid chromatography (RP-IP DHPLC) technology. Therefore, under denaturing conditions, due to the increased hydrophobicity of the biotin moiety attached to one of the strands, the retention time in HPLC of the two strands is different. So the separation of the ssDNA species from the PCR products is achieved through DHPLC. But this obviously is an expensive and instrument dependent method. In lambda exonuclease digestion method [31], a phosphorylated reverse primer is used in amplification. Then the phosphorylated strand is digested by lambda exonuclease and the remaining sense stranded is obtained. It has a drawback that incomplete digestion may lead to the contamination of the dsDNA in a reaction mixture. In size separation derived from unequal primers with chemical or structural modification method [32], reverse primer is designed with a chemical terminator or a GC-rich stem-loop structure at its 5’-end. So unequal strands of DNA could be created and subsequently separated on a denaturing urea–polyacrylamide gel. It can achieve a high recovery rate and purity of ssDNA. Currently, the most commonly used method for the isolation of ssDNA from dsDNA is magnetic separation with streptavidin-coated beads [33]. In the method, biotinylated reverse primer is used for PCR amplification. Biotinylated PCR products are immobilized onto streptavidin-coated beads and the unmodified ssDNA is rapidly separated from biotinylated strands by alkaline denaturation.

## 4. New Methods Derived from Cell-SELEX

With the applications of aptamer technology in the tumor cell detection and treatment, cell-SELEX technology has gradually developed, and the range of targets was gradually expanded [34]. Currently a variety of new screening methods based on cell-SELEX have emerged to improve the success rate of aptamer screening. Here we provide a brief description of some of new cell-based SELEX methods.

### 4.1. TECS-SELEX

In 2005, Cerchia et al. [35] reported for the first time that screening of aptamers specifically inhibit the receptor tyrosine kinase RET (Rearranged during Transfection) via the RET over-expression cell line. In the study, PC12/MEN2A cells that over-express the human RET were used as targets. The identified sequences bound to target cells with apparent *K*_d_ values ranging from 30 to 70 nm, while showed no tight binding to parental PC12 cells. Moreover, the aptamer obtained by the whole-cell SELEX strategy not only recognized the extracellular domain of RKT, but also blocked RET downstream signaling and subsequent molecular and cellular events. Next, Ohuchi et al. [36] developed a novel SELEX procedure named TECS-SELEX, in which a cell-surface displayed recombinant protein was directly used as the selection target. Using this method, they isolated RNA aptamers against transforming growth factor-β (TGF-β) type III receptor expressed on Chinese hamster ovary (CHO) cells. One of the RNA aptamers has dissociation constant at 1 nM range and competed with TGF-β to bind to the cell surface receptor in vitro. The development of TECS-SELEX provides a useful, novel method to isolate aptamers against any cell surface proteins of interest and especially useful when the purified protein target cannot be easily obtained.

### 4.2. FACS-SELEX

In 2010, Mayer et al. [21] published a detailed protocol based on fluorescence-activated cell sorting (FACS) to select aptamers that target to the subpopulations of cells. In this method, a fluorescently labeled aptamer library is incubated with the target cells. A FACS device is used to simultaneously differentiate and separate cells that bound to aptamers, which is rather sensitive, efficient and high-throughput. The bound aptamers are then eluted, purified and amplified. Cell membrane integrity is a prerequisite for the success of the SELEX process. Thus, those undergoing apoptosis or necrosis cells, which may take up nonspecifically binding nucleic acids in SELEX process, need to be eliminated. Conversely, FACS could exclude the impact of dead cells by setting a region of interest in a forward versus side scatter dot plot on membrane-intact cells, so that it can eliminate the false negative and improve the separation efficiency. The protocol provides a state-of-the-art approach for identifying aptamers that selectively target any cells under investigation. In 2014, Kim et al. [37] performed FACS-SELEX to isolate the aptamers against EpCAM—A transmembrane glycoprotein detected in most adenocarcinomas and cancer stem cells. The selected anti-EpCAM aptamer EP166 could distinguish cells expressing EpCAM from negative control cells.

### 4.3. 3D Cell-SELEX

3D cell-SELEX is a novel strategy to select specific nucleic acid ligands against spheroid cells in 3D cell culture to mimic the tissue microenvironment in vitro [22]. The 3D cell culture is performed by magnetic levitation method (MLM). This magnetic field is applied on top of the culture plate to promote magnetic levitation and generate levitated spheroid cells in the incubation period. Compared with the 2D-cell culture, the method makes the extracellular domains of membrane proteins more homogeneous exposure to aptamers and allows better cell-aptamer interaction. Integrated with a negative selection against a non-tumor cell line in the first round of 3D cell-SELEX, Aline lab showed that they obtained the aptamer A4 as a specific ligand to prostate tumor cells after nine selection cycles by 3D cell-SELEX, with dissociation constant in the nanomolar scale.

### 4.4. Hybrid-SELEX

Traditional cell-SELEX provides low aptamer enrichment efficiency as off-target surface biomarkers/molecules are co-expressed on the cells of interest. To overcome this obstacle, Hicke’s lab [38] introduced a hybrid-SELEX that combines the cell-based SELEX with purified protein-based SELEX techniques to develop Tenascin-C-specific RNA aptamers in 2001. In hybrid-SELEX, both purified proteins and cells bearing the same protein on their surface were used as targets. After a certain number of rounds of cell-SELEX, two additional rounds of selection with purified target protein, as a crossover SELEX experiment, were performed to enrich TN-C aptamer representation in the cell aptamer pools. Compared the screening results, two rounds of crossover selection on purified target protein improved the affinity by 50-fold of the cell-SELEX result pool. The first cell-SELEX process in hybrid-SELEX aimed to select aptamers against target in its native state on the surface of the cells. The following additional rounds of selection with purified target were to enrich the high-affinity aptamers, which were rare in cell aptamer pool [39].

### 4.5. Cell-Internalization SELEX

Cell-internalization SELEX is a cell-based selection process for identification and characterization of cell-internalizing RNA aptamers for delivering siRNA drugs into the cytoplasm of target cells. In the novel selection strategy, the main steps are recovering the internalized RNA sequences for amplification and discarding unbound or surface-bound RNAs in the iterative selection. Thereby it enriches RNAs that are internalized by the target cell. The principal obstacle to RNAi-based therapeutics is cellular uptake. Cell-internalizing RNA aptamers conjugated with siRNAs as a delivery tool is being proposed to address this problem [40]. In 2012, Giangrande’s lab successively obtained aptamers that internalize into cells that express specific cell surface receptors (e.g., HER2 or TrkB) [41]. As an example, aptamers specifically identified and internalized HER2-expressing cells were covalently linked to siRNAs targeting the anti-apoptotic gene, Bcl-2. They demonstrated that, when applied to HER2-expressing breast cancer cells, the HER2 aptamer-Bcl-2 siRNA conjugates selectively internalize into HER2(+)-cells and silence Bcl-2 gene expression. Moreover, Bcl-2 silencing sensitizes these cells to chemotherapy (cisplatin), suggesting a potential new therapeutic approach for treating breast cancers with HER2(+)-status [42]. These studies indicated that technology might promote the widespread use of RNA-based reagents for targeted therapeutic applications.

## 5. Applications of Cell-SELEX in Cancer Diagnosis and Therapy

In the past 20 years, aptamer has attracted much attention because of the high specificity, high affinity and promising application in medical diagnosis and disease treatment. Cell-SELEX facilitated the development of aptamer based diagnostic and therapeutic technology for cancer research. It can not only select aptamers specifically against particular designed biomarkers, but also unknown biomarkers on the surface of cancer cells.

### 5.1. Application of Cell-SELEX Aptamers in Cancer Diagnosis

Using cell-SELEX technology, Shangguan et al. identified a DNA aptamer targeting a T cell acute lymphoblastic leukemia cell line CCRF-CEM for acute lymphoblastic leukemia diagnosis. The selected aptamer sgc8c could specifically recognize leukemia cells in human bone marrow aspirates in real clinical specimens [43]. Therefore, sgc8c holds a great promise in developing specific molecular probes for cancer diagnosis. In 2010, Sefah et al. [44] developed a panel of DNA aptamers against colorectal cancer cultured cell lines DLD-1 and HCT 116 by cell-SELEX. The selected aptamers have high affinity and selectivity to identify specific biomarkers associated with colorectal cancers. Subsequently, on the basis of the previous studies, Suwussa et al. [45] created pattern recognition of different cancer cells using aptamer-conjugated magnetic nanoparticles (ACMNPs). In their study, aptamer sgc8c (target to CCRF-CEM cells), KDED2a-3 (target to DLD-1 cells) and KCHA10 (target to HCT 116 cells) were used separately to conjugate with dispersed magnetic nanoparticles, and became stable nanoassemblies for cancer cells detection and diagnosis. The specificity and sensitivity of the method were demonstrated by detection in cell mixtures and complex biological media, including fetal bovine serum, human plasma, and whole blood. sgc8c-ACMNPs could successfully detect in mixtures with the ratio between target and non-target cells was as low as 1:100. KDED2a-3 and KCHA10-ACMNPs also demonstrated strong specificity to their targets. Simultaneously, none of these ACMNPs had any interaction with the normal cell line. This strategy for pattern recognition shortened the incubation time and increased specificity in unpurified native samples. These proof-of-concept studies of magnetic relaxation switches (MRSw) certificated that it was a practical way to detect cancer cells and comprehensive cancer cell profiling. Once bound to nanoparticles, the aptamers could function to target cells and provide a multivalent effect. Furthermore, a new cellular molecular profile can be created through an array of ACMNPs to help clinicians accurately identify cancer cells at the molecular and single-cell level. All these advantage, as well as the simple operation of magnetic relaxation instrument, will make ACMNP-based nanosensors potential approaches to early diagnosis and effective screening of cancer.

TTA1, AS1411 and MUC-1 are more successful aptamers that were reported to specifically bind to cancer cells or cancer tissues [46,47,48]. Aptamer TTA1 was selected to bind the extracellular matrix protein, tenascin-C, of cancer cells. Aptamer AS1411, identified by antiproliferation selection, binds to the nucleolin in the plasma membranes of cancer cells. Aptamer MUC-1 selected by protein-SELEX targets mucin (MUC-1), which is highly expressed by the majority of human adenocarcinomas. In 2009, Kang et al. [49] conjugated these three aptamers (TTA1, AS1411 and MUC-1) to quantum dots respectively, and to demonstrate multiplex detection of cancer cells using quantum-dot (QD)-conjugated aptamers. For confocal microscopic analysis of multiplex imaging of cancers, healthy and cancer cell lines were incubated with each QD-conjugate. QDAS1411 showed strong fluorescence activity on cellular membranes in HeLa, C6, PC3 and NPA cell. QD-TTA1 was clearly visualized at 605 nm on C6 cells, but it showed very weak fluorescence signals with PC3, HeLa and NPA cell lines. QD-MUC-1 showed relatively higher fluorescence signals in the membrane of C6 and HeLa cell lines than other cancer cells, as nucleolin, tenascin-C, and mucin proteins had different cellular expression in different cancers. Experiments proved these probes could detect and differentiate different types of particular cancer cells and produce a visible fluorescence signal in the presence of target cells. It can be determined that the QD-aptamer conjugate offers a promising tool to assess the diverse genetic expression in a single cell by the demonstration of different colors. However, the multiplex imaging approach must solve problems associated with the biosafety of QDs and biostability of aptamers prior to clinical application.

HER2 belongs to tyrosine kinase receptor and the epidermal growth factor receptor (EGFR or ErbB) family. The overexpression of HER2 is related to many cancers, including ovarian, lung, gastric and oral. Therefore, the monitoring of HER2 expression is conducive to the early diagnosis of cancer [50]. Javed et al. [51] developed a new in vitro assay to detect human epidermal growth factor receptor 2 (HER2) protein in 2015. The method was based on affinity dissociation of carbon nanotube (CNT)-wrapped anti-HER2 ssDNA aptamers. Firstly, they selected an anti-HER2 ssDNA aptamer (H2) using cell-SELEX. Then the selected anti-HER2 aptamer was packed around CNTs (coupled with magnetic microbeads previously) by physical wrappings. The thus-formed magnetic microbeads coated with uniform layers of CNTs were isolated by applying an external magnetic field. The magnetic nature of MBs allowed extensive washing of MB–CNTs for the effective removal of unattached CNTs and thereby minimized the surfactant effect. The high affinity and specificity of aptamers to HER2 protein on tumor cells enables the sensitive and accurate diagnosis of cancers with HER2 overexpression. The results demonstrated that the developed assay can be an effective approach in detecting native forms of disease biomarkers in free solutions or in biological samples, for accurate diagnosis.

Furthermore, aptamers targeting cancer-specific proteins, such as vascular endothelial growth factor (VEGF) [52], pigpen [53], prostate-specific membrane antigen (PSMA) [54], and receptor tyrosine kinase (RTK) [55], have been developed and studied. Cell-SELEX technology not only can generate aptamers that can recognize such unique features with very high affinity and specificity, but can discover molecular signatures of the cancer cells easily. With the specificity and high-recognition, aptamers as the new probes can correctly distinguish different cell types and even subtypes of cancer cells. Also, in comparison to antibody-based techniques, aptamer-based specific probe is less labor-intensive and more cost-effective. More importantly, the aptamers probes recognize the targets at their native state to create a true molecular profile of the disease cells, which is important in clinical application. In addition, the ease of site-specific chemical modification of aptamers makes it possible to conjugate to gold nano-particles or quantum dots for use in colorimetric or fluorescence detection of cancer cells, and aptamers also can conjugate to the gold nanorod for the enhancement of the signal. These devices are so high sensitivity with aptamers that they can be applied for the early diagnosis of cancer when the concentration of cells is relatively low [56]. Therefore the use of a panel of probes has a clear advantage over the single biomarker-based assays in clinical practice, providing much more information for accurate disease diagnosis and prognosis.

### 5.2. Application of Cell-SELEX Aptamers in Cancer Therapy

Aptamers generated via cell-SELEX can specifically recognize cell-surface biomarkers without prior knowledge of their molecular signature. Therefore, aptamers may also serve as drug payload or aptamer-functionalized nanoparticles helping drugs to get released in specific target regions, which can achieve drug targeted delivery. More importantly, due to better target-specific physical binding properties, aptamers have less off-target toxicity effects. The prostate-specific membrane antigen (PSMA) is considered to be an excellent prostate tumor cell marker expressed on the surface of prostate cancer cells. Lupold et al. [57] used the purified protein as the target, and identified an aptamer A10 that could recognize the extracellular domain of PSMA. Farokhzad et al. [58] synthesized a bioconjugate with docetaxel (Dtxl)-encapsulated nanoparticles and PLGA-b-PEG connected to RNA aptamers A10 for targeted delivery. They examined its efficacy for targeted delivery of Dtxl to prostate cancer cells and found that the conjugate is capable of specifically binding to tumor cells with PSMA over-expression, and then engulfed by the tumor cells to exert cellular toxicity and achieve targeted therapy. The targeted therapy also showed a significant anti-tumor efficacy and reduced toxicity in vivo in the nude mouse model than using Dtxl only [59]. In 2013, Taghdisi et al. [60] synthesized PEG-Apt-Epi complex to achieve targeted delivery of Epirubicin to cancer cells by PEGylated A10 aptamer. Epirubicin (Epi), an anthracycline, is one of the main chemotherapy agents in the treatment of a variety of tumors. Flow cytometry analysis and MTT assay showed that PEG-Apt-Epi complex delivery system was able to specifically deliver and internalize Epi to LNCaP cells, and thereby this system can reduce cytotoxic effects of Epi by targeted delivery.

In addition, aptamer-based delivery of drugs including chemotherapy drugs such as doxorubicin, docetaxel, daunorubicin and cisplatin, toxins such as gelonin, and various photodynamic therapy agents, as well as a variety of small interfering RNAs have been reported [61]. These reports demonstrated the potential utility of nanoparticle-aptamer bioconjugates for cancer therapeutic application. Aptamer-based delivery may enable the transport of drugs across a range of biological barriers including epithelial and endothelial, and facilitate the delivery of drugs to intracellular sites of action, enhancing the efficacy and safety of therapeutics. With the development of aptamer selection technologies and nanomedicine, aptamer-functionalized nanoparticles are being explored as promising platforms for targeted therapeutic and we expect it to advance from preclinical into clinical development for further evaluation in the continuous efforts.

Meanwhile, aptamers with the efficient targeting ability to cancer cells and tissues not only provides a promising way to delivery drug, but also can be used as an anti-cancer drug for cancer therapy. Up to date, several aptamers are evaluated in the clinical trials for treatment of different types of cancers. The most frequently studies aptamer for cancer treatment is AS1411. AS1411 is an unmodified guanosine rich 26-mer DNA strand. It binds to the external domain of nucleolin, which is a protein over-expressed on the surface of cancer cells and responsible for survival, growth, and proliferation of cells. More recently, AS1411 has now been tested in several cancer cell lines, including prostate, breast, lung, pancreatic, renal cell carcinoma, ovarian, cervical, colon and so on [62]. It also displayed anti-proliferative activity in almost every cancer cell type that was tested in vitro. However, the exact mechanism for the activity of AS1411 was not totally understood. The phase I clinical study of AS1411, which was completed in 2006, found that AS1411 can specifically target nucleolin without serious toxicity, making it the first in-human and the first in-class anticancer aptamer drug. Phase II clinical trial for acute myeloid leukemia found AS1411 has therapeutic efficacy to acute myeloid leukemia patients. However, in phase II, evaluation for renal cell carcinoma showed only one patient had a response to treatment during 35 patients who were enrolled and treated [63].

Additional anti-cancer aptamers are studied in the preclinical setting (Table 1). These aptamers share some common mechanisms with anti-cancer effects. One mechanism is to block the signaling pathways by inhibiting kinases, phosphatases, or carboxypeptidases, etc., to stop downstream activation and signaling for tumor growth, for example RET aptamer D4. Aptamer D4 could not only recognize the extracellular domain of RET, but also inhibit RET phosphorylation and block RET downstream signaling and subsequent molecular and cellular events [35]. Aptamers that have anti-tumor activity could be promising prognostic tools in cancer therapy. Another way is to bind to proteins that have a close connection with tumor development. At present, most aptamers for cancer therapy in research function by inhibiting target functional membrane proteins. For example, CD44 protein is responsible for the migratory ability of cells. Anti-CD44 DNA aptamer can form complexes with CD44 protein to inhibit the migration of the breast cancer cells [64]. The potential therapeutic efficacy of the aptamers will be evaluated in vivo in subsequent studies. In the meanwhile, more aptamers with promising anti-tumor efficacy in preclinical studies are expected to be evaluated in clinical trials to facilitate the development of aptamer based drug in cancer therapy in the near future.

## 6. Future Perspectives

Aptamers have been widely used in biomarker discovery and detection, cancer imaging, cancer therapy and several other fields based on cell-SELEX technology [76]. As recognition elements, aptamers have several advantages, including efficient and cost-effective chemical synthesis, easy and controllable modification with functional moieties to meet various clinical requirements, large-scale commercial production, nontoxicity and limited immunogenicity [77,78,79]. Cell-SELEX is also a promising technology because it selects aptamers against whole live cells without prior knowledge of resident proteins on the cell surface. Cell-SELEX aptamer is an ideal tool for preferential binding to diseased cells, especially cancer cells. More importantly, cell-SELEX technology paves the way for the selection of tumor- and cell-specific aptamers that target distinct subpopulations of cells in heterogeneous composite mixtures of cells that are present in primary tissues or body fluids. This method may also be a first step toward the use of aptamers for individualized diagnostic and medical applications because this selection process will be accessible to clinical laboratories for the assessment of high-affinity and specific cell-targeting agents, which are promising diagnostic or biomedical research tools, and thus is a useful alternative to antibody-based assays.

However, the development of aptamers derived from cell-based selection for cancer diagnosis and therapy is still in the early stages. There are several challenges that need to be addressed before it is applicable. First, to design and engineer nucleic acid aptamers as chemical antibodies for application in more disease states, more aptamers need to be screened. Although much effort has been made for developing novel, automated, or high-throughput systems SELEX techniques, it is still difficult to achieve in cell-based selection. Tumor cells have different species (including subtypes) and usually show complex molecular characteristics. In order to really filter the aptamer molecular fingerprint that may indicate different stages of the disease and different subtypes of cancer, SELEX technology still needs further research in terms of screening and identification of tumor markers, such as screening methods, labeling technique and tracing. In addition, the structures, folding patterns, binding affinities, and regulation of protein and/or cell functions of aptamers need to be explored. Pharmacokinetics, toxicity and off-target effects also remained to be resolved [80].

## Figures and Tables

**Table 1:** Aptamers specifically targeting cell surface proteins used in cancer therapy.

Target	Method	Aptamer	Applications	Year	Reference
RET	cell-SELEX	RNA	Metastatic breast cancer, gastrointestinal stromal tumors and non-small cell lung cancer therapy	2005	[35]
PTK7	cell-SELEX	DNA	Acute lymphoblastic leukemia therapy and diagnosis	2006	[10,43]
Immunoglobin Heavy Mu Chain (IGHM)	cell-SELEX	DNA	Burkitt lymphoma diagnosis and therapy	2007	[14,65]
O-Glycan-Peptide Signatures	cell-SELEX	DNA	Targeted delivery by conjugation with chlorine e6 (Ce6) for epithelial cells cancer therapy	2009	[66]
AXL	cell-SELEX	RNA	human glioma cell cancer therapy	2009	[67,68]
CD16α (FcγRIIIα)	hybrid-SELEX	DNA	Cancer immunotherapy via mediating cellular cytotoxicity	2011	[39]
HER2	cell-SELEX	DNA	HER2 positive breast cancer therapy and diagnosis	2011	[69]
Epithelial Cell Adhesion Molecule (EpCAM)	Cell-SELEX	RNA	Novel targeted nanomedicine and molecular imaging agents for cancer theranostics	2011	[70,71]
CD133	cell-SELEX	RNA	Cancer stem cell targeted therapeutics and molecular imaging.	2013	[27]
Alkaline Phosphatase Placental-Like 2 (ALPPL-2)	cell-SELEX	RNA	Pancreatic carcinoma diagnosis and therapy	2013	[72]
CD44/CD24	cell-SELEX	DNA	Breast cancer diagnosis and therapy	2014	[64]
The C-C Chemokine Receptor Type 5 (CCR5)	cell-SELEX	RNA	HIV therapy	2015	[73]
Cytokeratin 19	cell-SELEX	DNA	Metastatic hepatocellular carcinoma diagnosis and chemotherapy	2016	[74]
MRP1	hybrid-SELEX	RNA	Binding MRP1-expressing tumors and delivering the CD28 costimulatory signal to tumor-infiltrating lymphocytes.	2016	[75]
